# Mortality in farmed European eel (*Anguilla anguilla*) in Italy due to *Streptococcus iniae*

**DOI:** 10.1186/s13028-023-00669-y

**Published:** 2023-02-14

**Authors:** Teresa Pirollo, Alberto Perolo, Simone Mantegari, Ilaria Barbieri, Federico Scali, Giovanni Loris Alborali, Cristian Salogni

**Affiliations:** 1 Istituto Zooprofilattico Sperimentale della Lombardia ed Emilia Romagna “Bruno Ubertini”, Via Antonio Bianchi 7/9, 25124 Brescia, Italy; 2 A. I. A. - Agricola Italiana Alimentare S.p.A, Via Valpantena 18/G, 37142 Verona, Italy

**Keywords:** Bacterial septicaemia, European eel disease, Fish streptococcosis

## Abstract

**Background:**

Streptococcal infections are one of the main causes of fish disease. During the last decade, *Streptococcus iniae* has become one of the most important aquatic pathogens worldwide, causing high losses in marine and freshwater finfish. Clinical signs in farmed fish include loss of appetite, lethargy and grouping at the bottom of the tank. Gross changes comprise darkening of the skin and haemorrhage at the basis of fins and opercula. To date, *S. iniae* has been isolated from several wild and farmed fish species but never in the European eel (*Anguilla anguilla*). In Europe, eel production from aquaculture is around 4500 tonnes and Italy is the third largest producer. This communication represents the first report of an outbreak of *S. iniae* infection in European eels.

**Case presentation:**

The outbreak occurred at an eel farm in northern Italy between May 2021 and September 2021. The outbreak caused about 2% mortality per month, resulting in the loss of about 10% of the farmed fish. The diseased eels showed apathy, lethargy, inactivity and inappetence. In July 2021, three eels were necropsied. Necropsy revealed skin and branchial hyperaemia, a few skin ulcers, and diffuse peritoneal congestion with a few haemorrhagic-like spot lesions. Swab samples for bacteriology were taken from the kidneys, liver, spleen, and brain. Additionally, four eels were opened and swap samples as above were taken. All the investigated eels were found dead. Bacteriological examination revealed growth of *Streptococcus* spp. from all samples. Identification of *S. iniae* was done by biochemical characterization, the API20STREP microsystem, 16S rDNA sequencing, and MALDI-TOF. Antimicrobial therapy (oxytetracycline and erythromycin) was ineffective.

**Conclusions:**

This is the first report of *S. iniae* infection in the European eel**.** Although this may be an isolated outbreak, it is of concern due to the losses associated with this pathogen in fish worldwide and because the European eel is an endangered species. Due to the difficulties of controlling the disease with antimicrobials, it is advisable to plan other effective control measures, such as improving water quality and the environmental conditions, reducing fish density, improving biosecurity, and by using immunostimulants and, when possible, vaccines.

**Supplementary Information:**

The online version contains supplementary material available at 10.1186/s13028-023-00669-y.

## Background

The European eel (*Anguilla anguilla*) is a catadromous euryhaline fish characterized by a complex biological cycle involving marine, brackish, and freshwater habitats. Because reproduction in captivity is currently not possible, breeding still relies entirely on a wild sperm supply [1].

Italy is the third largest producer of farmed eels in Europe, comprising around 4500 tonnes in 2020 [2]. Italian eel aquaculture is either extensive (‘vallicoltura’) or intensive with open-circuits receiving well or surface water. Intensive farms are mostly located in northern Italy. Italian eel production has been steadily decreasing from 1000 tonnes per year in 2014 to 700 in 2020 [2, 3]. This reduction is mainly due to a decline in the wild population (elvers and adults), which is subjected to habitat loss, pollution, migration barriers, exotic fish invasions and climate change [4]. For this reason, Italy is committed to protecting the eel population following the measures for stock recovery and management plans established by the European Council Regulation 1100/2007 [5].

Farmed eels are susceptible to several diseases, mostly cause by parasites or bacteria [6], which can lead to high mortality. Among bacterial infections, the most common disease is “red plague” [1], involving haemorrhagic skin lesions caused by different agents, such as *Vibrio* spp. (*V. anguillarum*, *V. vulnificus*) [1, 6], motile *Aeromonas* spp. (*A. hydrophila* complex, *A. sobria* complex, and *A. caviae* complex, *A. jandei*) [7], *Pseudomonas anguilliseptica* [8], *Edwardsiella tarda* [9] and *Edwardisiella anguillarum* [10]. Other bacteria, such as flavobacteria, are commonly responsible for necro-ulcerative skin lesions [11]. Diseases caused by cocci, such as fish streptococcosis, are frequent in other fish species but are rarely reported in eel [12]. Fish streptococcosis is a widespread systemic disease caused by Gram-positive streptococci-shaped bacteria. Aetiologic agents involved in fish streptococcosis can be divided according to temperature into cold-water pathogens (mortality below 15 °C), such as *Vagococcus salmoninarum* and *Lactococcus piscium*, and warm-water pathogens (mortality above 15 °C), such as *Lactococcus garvieae*, *Streptococcus agalactiae*, *Streptococcus parauberis*, and *Streptococcus iniae* [12, 13]. These bacteria can cause economically impactful diseases in freshwater species such as rainbow trout (*Oncorhynchus mykiss*), tilapias (*Oreochromis* spp.) and sturgeons (*Acipenser* spp.) as well as saltwater species such as European seabass (*Dicentrarchus labrax*), turbot (*Psetta maxima*), barramundi (*Lates calcalifer*) and seriola (*Seriola* spp.) [12, 14–19].

In this case report, we describe an outbreak of septicaemic infection in European eel associated with severe clinical signs and mortality that occurred on a farm in northern Italy from May to September 2021. *Streptococcus iniae* was isolated from all samples collected from seven eels during the outbreak. This is the first evidence of *S. iniae* causing disease in European eel.

## Case presentation

A group of European eels (weight 700–1000 g) in an inland intensive farm showed signs of disease characterized by apathy, inappetence, crowding in the corners of the tanks and visible ulcerative skin lesions in the ventral and jugular areas. The farm water was supplied by a river and the eels were raised in concrete raceways, receiving surface water that can reach a temperature of 25–26 °C during summer.

Recurrent episodes of increased mortality and eels showing similar clinical signs had been observed during summer periods since 2019. Mortality started in May when the temperature of the water rose above 18 °C. Between July and August, the water reached the maximum temperature (26 °C) and mortality lasted until September when the temperature had dropped below 18 °C again. The monthly mortality rate during the outbreak remained low and stable, around 2%, resulting in the loss of approximately 10% of the farmed fish. These cause of the episodes of disease were not investigated until the summer 2021. This study reports the findings of this investigation.

Twenty-eight swabs from the brain, kidney, liver and spleen of seven recently deceased eels were aseptically collected for bacteriological examination. In addition, routine anatomopathological and parasitological (wet mounts of skin, gills, and intestine) examinations were performed for three of these subjects. At the time of sampling (July 2021), the stocking density was 10–15 kg/m^3^ and the water parameters were: temperature 25 °C, pH 7.4, hardness 10 °F. Unionised ammonia, nitrite and nitrate were below the threshold considered harmful to the species at that age which are 0.1, 30 and 500 mg/L, respectively [20].

**Fig. 1:**
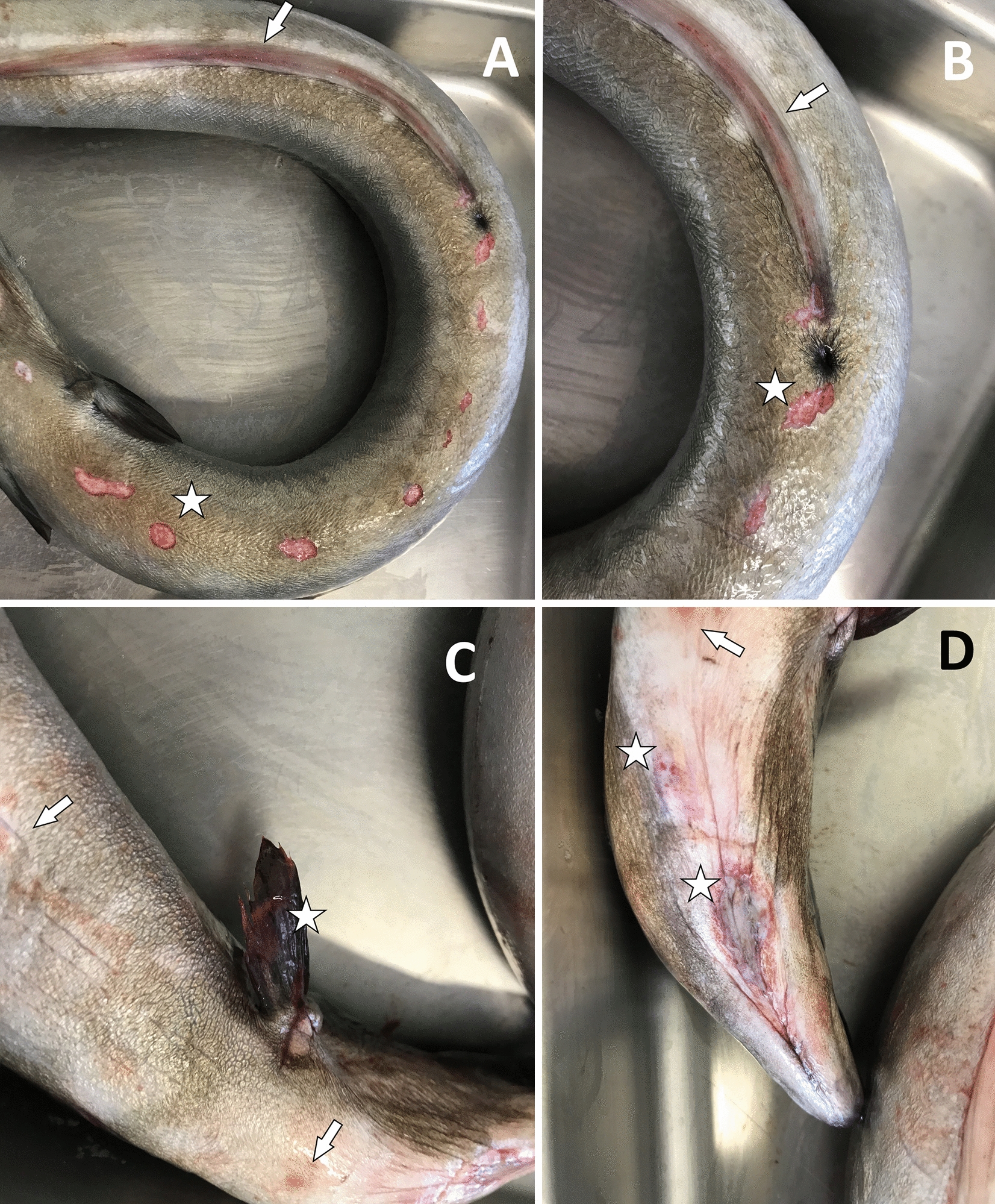
External examination of one of the European eels (*Anguilla anguilla*) involved in the disease outbreak. **A** Skin ulcers (an example highlighted with a star) and reddening of the anal fin (arrow). **B** Detail of **A** with skin ulcer example (an example highlighted with a star) and reddening of the anal fin (arrow). **C** Reddening of the skin (arrows) and ulceration of the pectoral fin (star). **D** Reddening of the skin (arrows) and ulcers (stars)

Necropsy revealed a good nutritional status of the eels, but they had a diffuse redness of the skin and fins (Fig. 1). In two eels, a few focal ulcerative skin lesions were found in the ventral and jugular areas (Fig. 1). A smear of the lesions, after staining with 2% fuchsin for two minutes, allowed us to identify by microscopy the presence of bacteria that were classified according to their shape as flavobacteria. The wet mount of gill tissue showed the presence of organic debris, hyperaemia, and rare parasites of the genus *Dactylogyrus*.

**Fig. 2:**
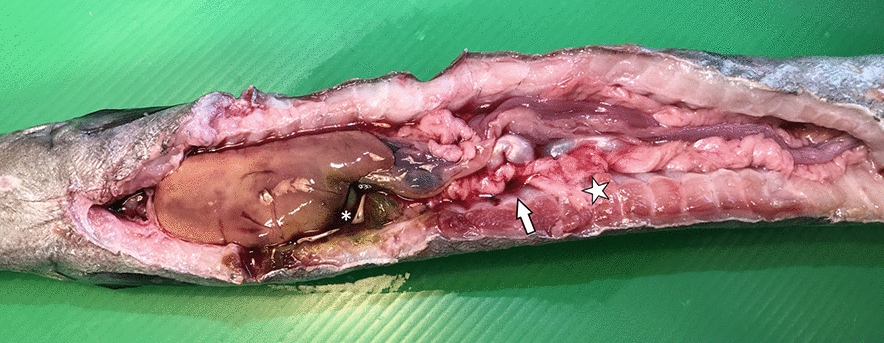
Appearance of the abdominal cavity viscera of one of the European eels (*Anguilla anguilla*) involved in the disease outbreak. Mild serosanguinous-looking exudate (arrow) and redness of the peritoneum (star). Note the empty gastroenteric tract and the enlarged gallbladder (asterisk)

Internal examinations did not reveal any severe lesions, but mild serosanguinous exudate and diffuse redness areas were present in the peritoneum (Fig. 2). The liver, kidney, and spleen appeared moderately hyperaemic but not enlarged. The gastroenteric tract was empty and the gallbladder enlarged (Fig. 2).

The swabs were inoculated onto Columbia blood agar (CA, 5% mutton red blood cells), tryptic soy agar (TSA), and thiosulfate citrate bile salt sucrose agar (TCBS) for bacteriological culturing. The plates were incubated under aerobic conditions at 22 °C for 48 h. After 2 days, there was abundant growth on both CA and TSA from all 28 samples, as characterized by whitish, umbonate microhaemolytic (up to 1 mm diameter) colonies and β-haemolysis, with appearance similar to that of the genus *Streptococcus*. A small number of other round-shaped haemolytic colonies were also present in three of the seven eels but only from liver and spleen samples. These colonies were identified as *Aeromonas* spp. and were classified as *Aeromonas sobria* complex after biochemical tests.

Colonies shaped like streptococci were selected from plates and re-inoculated onto TSA and CA and incubated for 48 h at 22 °C [21, 22]. The purified bacterial isolates were typed according to phenotypical (haemolytic activity, Gram stain), biochemical (macro method tests and miniaturized Analytical Profile Index (API) 20 STREP system), genotypic (16S rDNA sequencing), and proteomic [matrix-assisted laser desorption ionization-time of flight (MALDI-TOF) mass spectrometry analysis] characteristics.

Gram staining revealed Gram-positive cocci arranged in short chains or pairs. The biochemical tests identified the bacterial species as *S. iniae*. Biochemical tests were performed to differentiate colonies of *Streptococcus* spp. as follows: growth on CA at different temperatures (20, 37 and 42 °C), in a saline environment (TSA 6.5% NaCl), and on MacConkey agar and motility medium, the oxidase test and catalase test, arginine decarboxylation, esculin hydrolysis, indole production, urease, and acidification of sugars (mannitol, galactose, sorbitol, lactose, sucrose, inulin, trehalose) [18, 23, 24]. The results of the biochemical tests are shown in Table 1, with the field strain compared to two other reference strains [15, 18].

**Table 1:** Phenotypic characterization of *Streptococcus iniae* isolates from European eel (*Anguilla anguilla*) (wild strain) compared with two reference strains

	ATCC 29178 [12]	CIP 102508 [13]	Wild strain
Gram stain	+	+	+
Cell morphology	Cocci	Cocci	Cocci
Motility	−	−	−
Catalase test	−	−	−
Oxidase	−	−	−
Growth on MacConkey agar	−	−	−
Growth on (TSA^a^ 6.5% NaCl)	−	nd	−
Growth at 42 °C	−	nd	−
Growth at 37 °C	nd	nd	+
Pyrrolidonyl arylamidase	nd	+	−
Voges–Proskauer test	nd	−	−
Hippurate hydrolysis	nd	−	−
Esculin hydrolysis	+	+	+
α-Galactosidase	−	−	−
β-Glucuronidase	−	+	−
β-Galactosidase	+	−	−
Alkaline phosphatase	nd	+	+
Leucine aminopeptidase	nd	+	+
Arginine dihydrolase	nd	+	−
Acid from amidon	nd	+	+
Acid from arabinose	nd	−	−
Acid from galactose	+	nd	+
Acid from glycogen	nd	+	+
Acid from inulin	nd	−	−
Acid from lactose	nd	−	−
Acid from mannitol	nd	+	+
Acid from raffinose	−	−	−
Acid from ribose	nd	+	−
Acid from sorbitol	nd	−	−
Acid from sucrose	+	nd	+
Acid from trehalose	nd	+	+
Arginine decarboxylation	nd	+	+
Esculin degradation	nd	+	−
Indole production	nd	−	−
Urease	nd	−	−

Phenotypical comparison of the *Streptococcus iniae* strain isolated from European eel (wild strain) with two reference strains (ATCC 29178 and CIP 102508). Positive tests are indicated with ‘+’, negative with ‘−’, and not determined with ‘nd’

^a^Tryptic soy agar

Despite some variations, the biochemical profile obtained from the API 20 strep (code 4063116) agrees with other previous results derived from *S. iniae* strains isolated from other species [25]. Nevertheless, the code identifies more than one taxon because a biochemical profile, specific to *S. iniae* only, does not yet exist in any of the automated devices [12, 25].

In parallel with the biochemical analysis, the bacterium was identified using 16S rDNA sequencing and MALDI-TOF.

Partial sequencing of 16S ribosomal DNA (rDNA) was performed by means of MicroSEQ 500 16S rDNA Bacterial Identification System (Applied Biosystems) according to Patel et al. [26]. Briefly, the bacterial suspension was inactivated and the DNA was extracted in PrepMan Ultra Sample Preparation Reagent (Life Technologies) by lysis boiling at 98 °C for 10 min. A 500-bp 16S rDNA fragment was then amplified from the 5′ end of the gene in a reaction volume of 30 μL (15 μL of MicroSEQ PCR master mix and 15 μL of bacterial extract) using MicroSEQ 500 rDNA PCR kit (Applied Biosystems). The PCR conditions were as follows: 10 min of initial denaturation at 95 °C, followed by 30 cycles of annealing of 30 s at 95 °C, 30 s at 60 °C, 45 s at 72 °C, and a final 10-min extension at 72 °C. The amplicon was then purified using a QIAquick PCR purification Kit (Qiagen) according to the manufacturer’s recommendations. Forward and reverse sequencing reactions were performed on an amplified product by MicroSEQ 500 rDNA sequencing kit (Applied Biosystems) following the manufacturer’s instruction. The sequencing reactions consisted of 13 μL of MicroSEQ sequencing mix and 7 μL of purified amplified product. Thermal cycling conditions were as follows: 25 cycles of annealing of 10 s at 96 °C, 5 s at 50 °C and 4 min at 60 °C. Sequencing reactions were purified with BigDye XTerminator Purification Kit (Applied Biosystems) according to the manufacturer’s instructions. The sequence reactions were separated by capillary electrophoresis onto a 3500xl Genetic Analyzer (Applied Biosystems) according to standard automated sequencer protocols. A BLAST search of the 16S rDNA sequence showed 99.43% homology with an *S. iniae* GX005 (GenBank Accession no. CP032401), a bacterial strain isolated from the olive flounder (*Paralichthys olivaceus*), and 99.24% homology with *S. iniae* ATCC 29178 (GenBank Accession no. NR_025148.1). *Streptococcus iniae* was identified at 99.90% by MALDI TOF, Vitek MS Plus, Biomerieux, France (Additional file 1).

The isolated strain of *S. iniae* was tested for antimicrobial susceptibility by disc diffusion method on Mueller Hinton Agar (Oxoid, Italy) [27]. The test was performed using the Mueller Hinton solid medium and added mutton red blood cells (5%), with seeding of a bacterial suspension at a turbidity of 0.5 McFarland and incubation under aerobic conditions for 48 h at 22 °C, following the standard protocols of the Clinical and Laboratory Standard Institute (CLSI) [28]. Since breakpoints for the European eel are not available, we report the test results with those for mammals, according to CLSI guidelines [29]. The microorganism was susceptible to amoxicillin + clavulanic acid (30 µg), ampicillin (10 µg), cephalothin (30 µg), ceftiofur (30 µg), enrofloxacin (5 µg), erythromycin (15 µg), penicillin (10 µg), tetracycline (30 µg), and thiamphenicol (30 µg). Intermediate sensitivity to florfenicol (30 µg) and trimethoprim + sulfonamide (25 µg) was observed and resistance to nalidixic acid (30 µg), kanamycin (30 µg), oxacillin (1 µg), and pirlimycin (2 µg).

Antimicrobial therapy was implemented using medicated food. A first treatment cycle was carried out with oxytetracycline at 75 mg/kg body weight (BW) for 10 days but did not provide any appreciable results. A second treatment was then performed with erythromycin at 70 mg/kg BW for 10 days which was unsuccessful too. Indeed, the eels remained anorexic during the therapies likely resulting in an inadequate medicated feed intake. The clinical signs and related mortality disappeared completely when the water temperature dropped naturally below 18 °C.

## Discussion and conclusions

The identification of *S. iniae* in aquatic animals was originally done in 1976 from the subcutaneous lesions of a captive freshwater dolphin, *Inia geoffrensis*, in San Francisco, California, USA [23]. Since then, the bacterium has also been found in several fish species, becoming an important pathogen in the last decade and causing massive losses in wild and farmed fish worldwide [16]. Its rapid spread is due to its poor host species specificity; in fact, at least 27 species of fish have been found to be susceptible to *S. iniae* [12]. Nevertheless, *S. iniae* infection in European eel has not been reported.

The capsule of *S. iniae* is a major virulence factor that provides resistance to the bactericidal activity of phagocytes while stimulating a prolonged inflammatory response. In severe cases, the infection can lead to septicaemia [12, 16, 30]. Lesions can comprise gill rot, ascites and haemorrhages at the operculum, around the mouth, at the base of the fins and on viscera [12, 31]. The progression of streptococcosis in fish may vary depending on the virulence of the isolate, route of infection, the host species affected, age of the fish, as well as on environmental and water quality factors [12]. Not only the clinical but also the epidemiological features of the *S. iniae* outbreak described in this case shared similarities to those reported in other species farmed in warm waters. Indeed, the outbreak involved only adults and was influenced by water temperature [12].

The introduction and spread of *S. iniae* infections can occur in several ways. In particular, by horizontal transmission, with incoming water, with the persistence of the agent in the sediment, by faecal transmission, with the introduction of carrier fish and through cannibalism (dead or dying infected individuals) [12, 31, 32]. In our case, the most likely source of introduction was incoming water. The farm was supplied with surface water, which is a known risk factor especially when infected wild fish are present near the farm [33–35].

In this case, the infection of *S. iniae* in European eel may be considered a consequence of environmental stressors such as intensive farming conditions and low water quality, especially due to high water temperature during the summer season, which is a major risk factor for *S. iniae* infection [36]. Several studies reported a relationship between water temperature and *S. iniae* infections [12, 17, 31]. For example, Bromage and Owens identified a mortality peak at 25–27 °C in infected barramundis [36]. In addition to high temperatures, crowded conditions in which the eels were farmed may also have favoured the spread of the pathogen. External parasites and damaged skin have an immunosuppressive effect on fish that could facilitate the onset and increase the severity of the disease [37]. We found parasites of the genus *Dactylogyrus* spp. in the gill tissue and the presence of shape-similar flavobacteria colonizing the skin ulcer, which may had increased the susceptibility of eels to infection with *S. iniae*.

*Aeromonas sobria* complex was isolated in this case too. Although there are no reported cases of co-infection between *S. iniae* and *A. sobria* in other species, several other bacterial co-infections have been reported in fish [38]. In particular, co-infections of *S. iniae* and another motile aeromonad (*A. hydrophila*) were recently described in Nile tilapia (*Oreochromis niloticus*) [39, 40]. In our case, even though a co-infection cannot be excluded, the most likely cause of the presence of *A*. *sobria* complex is contamination during sampling. Indeed, only a few colonies had grown from samples of a small number of subjects (three out of seven) and from only the liver and spleen. In contrast, *S. iniae* showed abundant growth from all 28 samples collected. Regardless of its role, *A. sobria* complex should not be considered the primary agent of the disease. In addition to the limited number of colonies grown, unlike *S. iniae*, *A. sobria* complex are bacteria that affects fish of all ages and without specific limitations in water temperature [41, 42].

Antimicrobials were successfully used to control streptococcal infection in other species, especially in cases of disease outbreaks [43–46]. On the other hand, antimicrobial therapy could fail because resistant strains are involved, the bacteria can survive within macrophages or sick fish may struggle to eat the medicated feed [12]. In our case, treatment failure could be mainly due to eels’ anorexia leading to an insufficient feed intake.

As an alternative to antimicrobials, vaccines and probiotics could be used to control and also prevent *S. iniae* infections. Several immunostimulants can increase the innate immune protection of fish. Recently, dietary intake of probiotics such as plant extracts, nutritional factors, polysaccharides, and cytokines has been reported to be effective immunostimulants in fish, although more in-depth species-specific studies are needed [47]. The use of commercial vaccines could be difficult because, although several formulations are available worldwide [12, 31, 47, 48], these are not available in Italy. Another challenge in implementing a vaccination plan is the lack of cross-protection against the different *S. iniae* strains [12]. Indeed, the disease has been observed in vaccinated fish after the introduction of new strains [12]. An autogenous vaccine produced from cultures of the farm’s isolates can represent an effective alternative to commercial products but the use of such vaccines could be limited by high costs [44, 47]. Furthermore, considering that in our case the farm is supplied with surface water, the efficacy of an autogenous vaccine could also be impaired by the introduction of new and different strains.

The presentation of this case has some limitations due to the unexpected isolation of *S. iniae*. For example, a histological examination could have provided a more precise insight into the pathology. Eel tissues are very delicate and by the time we became aware of the agent, it was too late to preserve tissues for histology. In the case of outbreaks with characteristics similar to those described in this report, we recommend performing a histological examination.

In conclusion, this case report highlights the appearance of *S. iniae,* an aquatic pathogen of global significance, in farmed European eel for the first time. This outbreak may represent an isolated case or, at any rate, a rare occurrence. Nevertheless, the European eel is considered critically endangered by the International Union for Conservation of Nature [49] and *S. iniae* may potentially pose another threat to wild populations, particularly when they are under stressful environmental conditions. Regarding farmed eels, our report emphasizes the importance of monitoring outbreaks that can occur on farms by performing proper diagnostic investigations. Considering both the difficulty of controlling the disease with antimicrobial therapy and the importance of reducing the use of antimicrobials in food-producing animals, it is advisable to plan other control measures effective against potential risk factors, such as improving biosecurity, water quality and environmental conditions as well as reducing fish density.

## Supplementary Information

**Additional file 1.** Main spectrum profiles (MSPs) of the *Streptococcus iniae* isolate showing intensity (Y-axis) as a function of the mass-to-charge ratio (m/z, molecular weight for a single positive charge; X-axis). Peaks with intensities greater than 2000 are labelled.

## Data Availability

The datasets and the isolates analysed during the current study are available from the corresponding author on reasonable request.

## References

[1] Elgendy MY, Kenawy AM, Noor El-Deen AE (2016). *Gyrodactylus anguillae* and *Vibrio vulnificus* infections affecting cultured eel, *Anguilla anguilla*. Comun Sci.

[2] Federation of European Aquaculture Producers. European aquaculture production report 2014–2020 (V1.1). 2021. https://feap.info/wp-content/uploads/2022/03/production-report-v1.1.pdf. Accessed 20 Dec 2022.

[3] Corbari L, Mezzani G, Rossi R, Cataudella S, Bronzi P (2001). Anguillicoltura. Acquacoltura Responsabile - Verso le Produzioni Acquatiche del Terzo Millennio.

[4] Aschonitis V, Castaldelli G, Lanzoni M, Rossi R, Kennedy C, Fano EA (2017). Long-term records (1781–2013) of European eel (*Anguilla anguilla* L.) production in the Comacchio Lagoon (Italy): evaluation of local and global factors as causes of the population collapse. Aquat Conserv Mar Freshw Ecosyst.

[5] Council E (2007). Council Regulation (EC) No 1100/2007 of 18 September 2007 establishing measures for the recovery of the stock of European eel. Off J Eur Union.

[6] Woo PTK, Bruno DW (2011). Fish diseases and disorders. Volume 3: viral, bacterial and fungal infections.

[7] Esteve C, Biosca E, Amaro C (1993). Virulence of *Aeromonas hydrophila* and some other bacteria isolated from European eels *Anguilla anguilla* reared in fresh water. Dis Aquat Organ.

[8] Andree KB, Rodgers CJ, Furones D, Gisbert E (2013). Co-Infection with *Pseudomonas anguilliseptica* and *Delftia acidovorans* in the European eel, *Anguilla anguilla* (L.): a case history of an illegally trafficked protected species. J Fish Dis.

[9] Alcaide E, Herraiz S, Esteve C (2006). Occurrence of *Edwardsiella tarda* in wild European eels *Anguilla anguilla* from Mediterranean Spain. Dis Aquat Organ.

[10] Shao S, Lai Q, Liu Q, Wu H, Xiao J, Shao Z (2015). Phylogenomics characterization of a highly virulent *Edwardsiella strain* ET080813T encoding two distinct T3SS and three T6SS gene clusters: propose a novel species as *Edwardsiella anguillarum* sp. nov.. Syst Appl Microbiol.

[11] Soares SMC, Walker A, Elwenn SA, Bayliss S, Garden A, Stagg HEB (2019). First isolation of *Flavobacterium psychrophilum* associated with reports of moribund wild European eel (*Anguilla anguilla*) in Scotland. J Fish Dis.

[12] Agnew W, Barnes A (2007). *Streptococcus iniae*: an aquatic pathogen of global veterinary significance and a challenging candidate for reliable vaccination. Vet Microbiol.

[13] El-Noby GA, Hassanin M, El-Hady M, Aboshabana S (2021). *Streptococcus*: a review article on an emerging pathogen of farmed fishes. Egypt J Aquat Biol Fish.

[14] Eldar A, Ghittino C (1999). *Lactococcus garvieae* and *Streptococcus iniae* infections in rainbow trout *Oncorhynchus mykiss*: similar, but different diseases. Dis Aquat Organ.

[15] Ortega C, García I, Irgang R, Fajardo R, Tapia-Cammas D, Acosta J (2018). First identification and characterization of *Streptococcus iniae* obtained from tilapia (*Oreochromis aureus*) farmed in Mexico. J Fish Dis.

[16] Pierezan F, Shahin K, Heckman TI, Ang J, Byrne BA, Soto E (2020). Outbreaks of severe myositis in cultured white sturgeon (*Acipenser transmontanus* L.) associated with *Streptococcus iniae*. J Fish Dis.

[17] Mugetti D, Colussi S, Pastorino P, Varello K, Tomasoni M, Menconi V (2022). Episode of mortality associated with isolation of *Streptococcus iniae* in Adriatic sturgeon (*Acipenser naccarii* Bonaparte, 1836) reared in Northern Italy. J Fish Dis.

[18] El Aamri F, Padilla D, Acosta F, Caballero MJ, Roo J, Bravo J (2010). First report of *Streptococcus iniae* in red porgy (*Pagrus pagrus* L.). J Fish Dis.

[19] Bromage ES, Thomas A, Owens L (1999). *Streptococcus iniae*, a bacterial infection in barramundi *Lates calcarifer*. Dis Aquat Organ.

[20] EFSA (2008). Scientific opinion of the panel on animal health and welfare on a request from the European Commission on animal welfare aspects of husbandry systems for farmed European eel. EFSA J.

[21] Borella L, Salogni C, Vitale N, Scali F, Moretti VM, Pasquali P (2020). Motile aeromonads from farmed and wild freshwater fish in northern Italy: an evaluation of antimicrobial activity and multidrug resistance during 2013 and 2016. Acta Vet Scand.

[22] Austin B, Austin DA (2016). Bacterial fish pathogens: disease of farmed and wild fish.

[23] Pier GB, Madin SH (1976). *Streptococcus iniae* sp. nov., a beta-hemolytic *Streptococcus* isolated from an Amazon Freshwater Dolphin, *Inia geoffrensis*. Int J Syst Bacteriol..

[24] Dodson SV, Maurer JJ, Shotts EB (1999). Biochemical and molecular typing of *Streptococcus iniae* isolated from fish and human cases. J Fish Dis.

[25] Buller NB, Cutts R, McCann E, Bishop J (2014). Biochemical identification tables. Bacterial and fungi from fish and other aquatic animals: a practical identification manual.

[26] Patel JB, Leonard DGB, Pan X, Musser JM, Berman RE, Nachamkin I (2000). Sequence-based identification of *Mycobacterium* species using the MicroSeq 500 16S rDNA bacterial identification system. J Clin Microbiol.

[27] Bauer AW, Kirby WM, Sherris JC, Turck M (1966). Antibiotic susceptibility testing by a standardized single disk method. Am J Clin Pathol.

[28] CLSI. Methods for antimicrobial dilution and disk susceptibility testing of infrequently isolated or fastidious bacteria, 3rd edition. CLSI guideline M45. Wayne: Clinical and Laboratory Standards Institute; 2015.

[29] CLSI (2018). Performance standards for antimicrobial disk and dilution susceptibility tests for bacteria isolated from animals.

[30] Chen D, Peng S, Chen D, Yang F, Liu J, Wang J (2020). Low lethal doses of *Streptococcus iniae* caused enteritis in Siberian sturgeon (*Acipenser baerii*). Fish Shellfish Immunol.

[31] Shoemaker CA, Xu D-H, Soto E (2017). *Streptococcus iniae* and *S. agalactiae*. Fish viruses and bacteria pathobiology and protection.

[32] Salati F, Woo TKP, Bruno WD (2011). *Enterococcus seriolicida* and *Streptococcus* spp. (*S. iniae*, *S. agalactiae* and *S. dysgalactiae*). Fish diseases and disorders. Volume 3: viral, bacterial and fungal infections.

[33] Colorni A, Diamant A, Eldar A, Kvitt H, Zlotkin A (2002). *Streptococcus iniae* infections in Red Sea cage-cultured and wild fishes. Dis Aquat Organ.

[34] Zlotkin A, Hershko H, Eldar A (1998). Possible transmission of *Streptococcus iniae* from wild fish to cultured marine fish. Appl Environ Microbiol.

[35] Piamsomboon P, Thanasaksiri K, Murakami A, Fukuda K, Takano R, Jantrakajorn S (2020). Streptococcosis in freshwater farmed seabass Lates calcarifer and its virulence in Nile tilapia *Oreochromis niloticus*. Aquaculture.

[36] Bromage E, Owens L (2009). Environmental factors affecting the susceptibility of barramundi to *Streptococcus iniae*. Aquaculture.

[37] Xu DH, Shoemaker CA, Klesius PH (2007). Evaluation of the link between gyrodactylosis and streptococcosis of Nile tilapia, *Oreochromis niloticus* (L.). J Fish Dis.

[38] Kotob MH, Menanteau-Ledouble S, Kumar G, Abdelzaher M, El-Matbouli M (2016). The impact of co-infections on fish: a review. Vet Res.

[39] Delphino MK, Leal CA, Gardner IA, Assis GB, Roriz GD, Ferreira F (2019). Seasonal dynamics of bacterial pathogens of Nile tilapia farmed in a Brazilian reservoir. Aquaculture.

[40] Puneeth TG, Pallavi B, Vilasini U, Kushala KB, Nithin MS, Girisha SK (2022). Large scale mortality in cultured Nile tilapia (*Oreochromis niloticus*): natural co-infection with *Aeromonas hydrophila* and *Streptococcus iniae*. Iran J Vet Res.

[41] Wahli T, Burr SE, Pugovkin D, Mueller O, Frey J (2005). *Aeromonas sobria*, a causative agent of disease in farmed perch, *Perca fluviatilis* L.. J Fish Dis.

[42] Dien LT, Ngo TPH, Nguyen TV, Kayansamruaj P, Salin KR, Mohan CV (2022). Non-antibiotic approaches to combat motile *Aeromonas* infections in aquaculture: current state of knowledge and future perspectives. Rev Aquac.

[43] Darwish AM, Hobbs MS (2005). Laboratory efficacy of amoxicillin for the control *of Streptococcus iniae* infection in Blue Tilapia. J Aquat Anim Health.

[44] Creeper JH, Buller NB (2006). An outbreak of *Streptococcus iniae* in barramundi (*Lates calcarifera*) in freshwater cage culture. Aust Vet J.

[45] Gaunt PS, Endris R, McGinnis A, Baumgartner W, Camus A, Steadman J (2010). Determination of florfenicol dose rate in feed for control of mortality in Nile Tilapia infected with *Streptococcus iniae*. J Aquat Anim Health.

[46] Stoffregen DA, Backman SC, Perham RE, Bowser PR, Babish JG (1996). Initial disease report of *Streptococcus iniae* infection in hybrid striped (sunshine) bass and successful therapeutic intervention with the fluoroquinolone antibacterial enrofloxacin. J World Aquac Soc.

[47] Mishra A, Nam GH, Gim JA, Lee HE, Jo A, Kim HS (2018). Current challenges of *Streptococcus* infection and effective molecular, cellular, and environmental control methods in aquaculture. Mol Cells.

[48] Heckman TI, Soto E (2021). *Streptococcus iniae* biofilm formation enhances environmental persistence and resistance to antimicrobials and disinfectants. Aquaculture.

[49] Pike C, Crook V, Gollock M. *Anguilla anguilla*. The IUCN red list of threatened species 2020: e.T60344A152845178. 2020. 10.2305/IUCN.UK.20202.RLTS.T60344A152845178.en. Accessed 05 July 2022.

