# Supplementary material for: Mortality in farmed European eel (Anguilla anguilla) in Italy due to Streptococcus iniae
Source: Acta Vet Scand. 2023 Feb 14;65:5. doi: 10.1186/s13028-023-00669-y (PMC9926715; doi:10.1186/s13028-023-00669-y)
Supplement: Supplementary file 1 — Additional file 1. Main spectrum profiles (MSPs) of the Streptococcus iniae isolate showing intensity (Y-axis) as a function of the mass-to-charge ratio (m/z, molecular weight for a single positive charge; X-axis). Peaks with intensities greater than 2000 are labelled. [file 13028_2023_669_MOESM1_ESM.pdf]

Mortality in farmed European eel (*Anguilla anguilla*) in Italy due to *Streptococcus iniae*

Teresa Pirollo, Alberto Perolo, Simone Mantegari, Ilaria Barbieri, Federico Scali, Giovanni Loris Alborali, Cristian Salogni

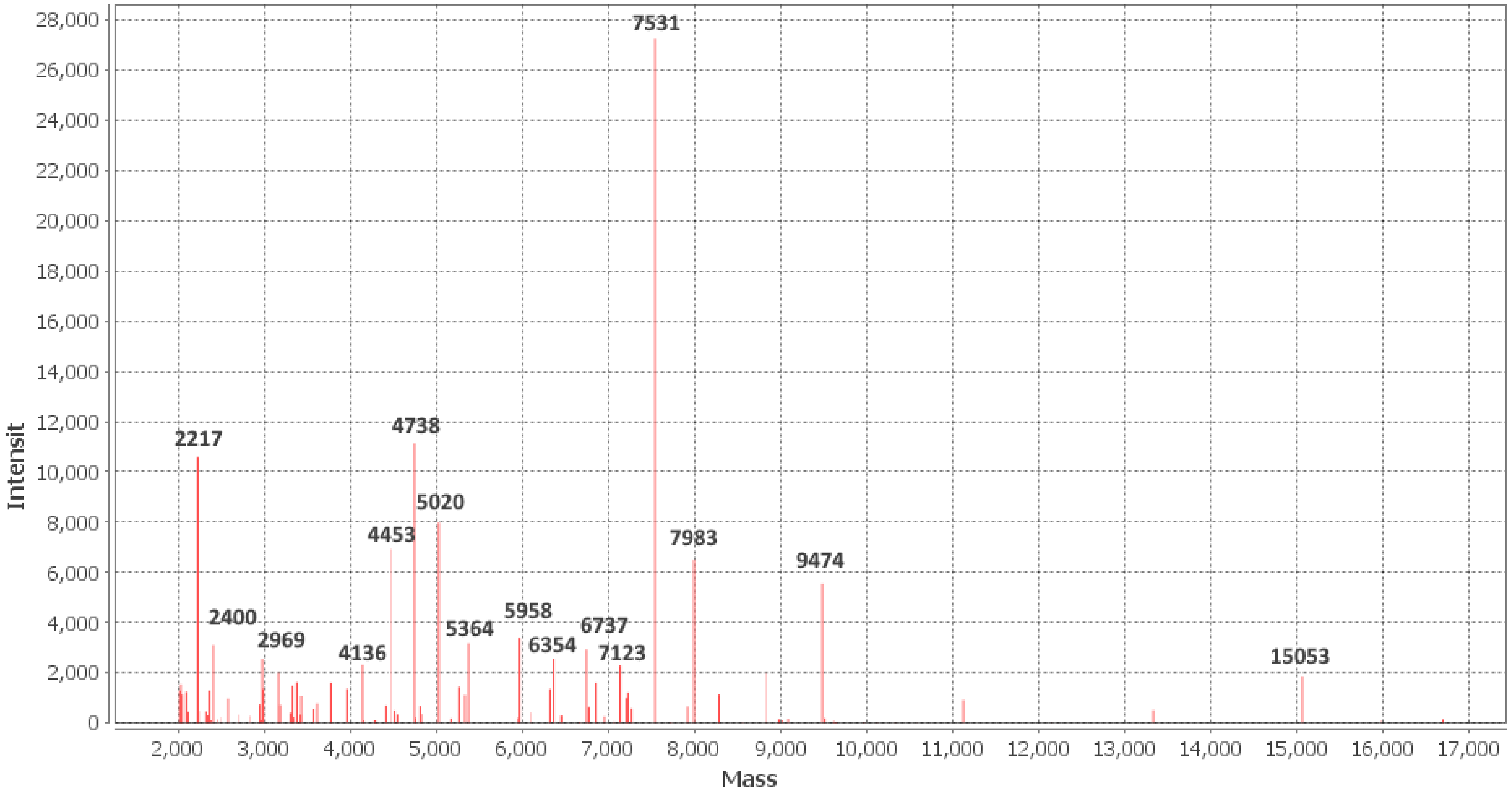

Additional File 1. Main spectrum profiles (MSPs) of the *Streptococcus iniae* isolate showing intensity (Y-axis) as a function of the mass-to-charge ratio (m/z, molecular weight for a single positive charge; X-axis). Peaks with intensities greater than 2000 are labelled.
